# MEP pathway-mediated isopentenol production in metabolically engineered *Escherichia coli*

**DOI:** 10.1186/s12934-014-0135-y

**Published:** 2014-09-12

**Authors:** Huaiwei Liu, Yang Wang, Qiang Tang, Wentao Kong, Wook-Jin Chung, Ting Lu

**Affiliations:** Department of Bioengineering, University of Illinois at Urbana-Champaign, Urbana, IL61801 USA; Institute for Genomic Biology, University of Illinois at Urbana-Champaign, Urbana, IL61801 USA; Energy and Environment Fusion Technology Center (E2FTC), Department of Energy and Biotechnology (DEB), Myongji University, Yongin-si, Gyeonggi-do 449-728 Republic of Korea; Department of Physics, University of Illinois at Urbana-Champaign, Urbana, IL61801 USA

**Keywords:** Isopentenol, MEP pathway, Entner-Doudoroff pathway, Pentose phosphate pathway (PPP), *Escherichia coli*, Metabolic engineering

## Abstract

**Background:**

Isopentenols, such as prenol and isoprenol, are promising advanced biofuels because of their higher energy densities and better combustion efficiencies compared with ethanol. Microbial production of isopentenols has been developed recently via metabolically engineered *E. coli*. However, current yields remain low and the underlying pathways require systematic optimization.

**Results:**

In this study, we targeted the *E. coli* native 2-methyl-(D)-erythritol-4-phosphate (MEP) pathway and its upstream glycolysis pathway for the optimization of isopentenol production. Two codon optimized genes, *nudF* and *yhfR* from *Bacillus subtilis*, were synthesized and expressed in *E. coli* W3110 to confer the isopentenol production of the strain. Two key enzymes (IspG and Dxs) were then overexpressed to optimize the *E. coli* native MEP pathway, which led to a significant increase (3.3-fold) in isopentenol production. Subsequently, the glycolysis pathway was tuned to enhance the precursor and NADPH supplies for the MEP pathway by activating the pentose phosphate pathway (PPP) and Entner-Doudoroff pathway (ED), which resulted in additional 1.9 folds of increase in isopentenol production. A 5 L-scale batch cultivation experiment was finally implemented, showing a total of 61.9 mg L^−1^ isopentenol production from 20 g L^−1^ of glucose.

**Conclusion:**

The isopentenol production was successfully increased through multi-step optimization of the MEP and its upstream glycolysis pathways. It demonstrated that the total fluxes and their balance of the precursors of the MEP pathway are of critical importance in isopentenol production. In the future, an elucidation of the contribution of PPP and ED to MEP is needed for further optimization of isopentenol production.

**Electronic supplementary material:**

The online version of this article (doi:10.1186/s12934-014-0135-y) contains supplementary material, which is available to authorized users.

## Background

The growing global demand for energy, continuing concerns about the environment and the increasing cost of fossil fuels have called for the development of new forms of energy [1,2]. The microbial production of bio-based fuels and chemicals from biomass has recently become as a promising, sustainable solution to this need [3,4]. Early efforts in biofuel production have focused on improving the yield of ethanol made from the fermentation of plant sugars, and have shown a great success [5,6].

Recently, microbial production of advanced biofuels, such as butanol and isobutanol, is being increasingly explored due to the facts that advanced biofuels have higher energy contents and, more importantly, are more compatible with existing engines and fuel distribution infrastructures [7–9]. One class of such advanced biofuels is isopentenols, including prenol and isoprenol, which have better combustion efficiencies than ethanol and better octane numbers that are more similar to that of gasoline compared with ethanol [10].

Isopentenol production using engineered microbes was first reported in 2007 by Withers and co-workers [11]. Through the enrichment of a library of genomic DNA from *Bacillus subtilis*, the authors discovered two genes (*yhfR* and *nudF*) that encode enzymes that function directly on the prenyl diphosphate precursors, isopentenyl pyrophosphate (IPP) and dimethylallyl pyrophosphate (DMAPP) and convert them to isopentenols. In 2012, Chou and Keasling reported another gene *nudB* which encodes an enzyme capable of converting prenyl diphosphate precursors to isopentenols [10]. Overexpression of these genes along with the introduction of a heterologous mevalonate-dependent pathway (MVA) in *E. coli* has resulted in successful isopentenol production [10–12]. In addition to the MVA pathway, the *E. coli* native MEP pathway also holds the potential to be exploited for isopentenol production. Indeed, several attempts have been made for successful biosynthesis of other forms of isoprenoids including taxadiene through the *E. coli* native MEP pathway [13–15]. Up to date, reported isopentenol production via the MVA pathway is much higher than that via the MEP pathway. On the other hand, there are advantages in using the MEP pathway: According to the stoichiometry and redox balance analysis by a previous study [16], MEP is energetically more balanced and theoretically more efficient than MVA in converting sugars or glycerol to isoprenoid. In addition, due to the inherent presence of the MEP pathway, it requires fewer heterologous genes to be introduced.

**Figure 1:**
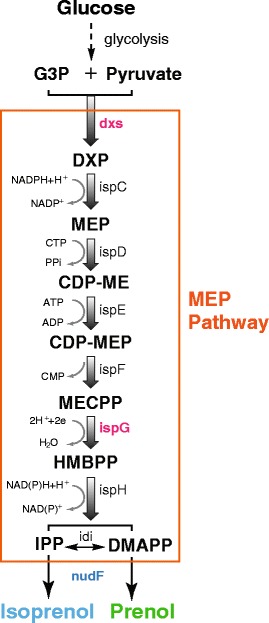
**The isopentenol biosynthesis pathway in engineered** ***E. coli.*** Pathway intermediates: G3P, glyceraldehyde-3-phosphate; DXP, 1-deoxy-D-xylulose 5-phosphate; MEP, 2-*C*-methyl-D-erythritol 4-phosphate; CDP-ME, 4-diphosphocytidyl-2-*C*-methyl-D-erythritol; CDP-MEP, 4-diphosphocytidyl-2-*C*-methyl-D-erythritol 2-phosphate; MECPP, 2-*C*-methyl-D-erythritol 2,4-cyclopyrophosphate; HMBPP, 1-hydroxy-2-methyl-2-(*E*)-butenyl 4-pyrophosphate; IPP, isopentenyl pyrophosphate; DMAPP, dimethylallyl pyrophosphate; DHAP, dihydroxyacetone 3-phosphate.

We were therefore motivated in this work to increase the isopentenol production of engineered *E. coli* by optimizing its inherent MEP pathway (Figure 1). We first synthesized two codon optimized genes, *nudF* and *yhfR* from *B. subtilis*, and expressed them in our expression host *E. coli* W3110 to confer its production of isopentenols including both prenol and isoprenol. We then overexpressed two key enzymes (IspG and Dxs) to optimize the native *E. coli* MEP pathway, leading to a significant increase in isopentenol titer. To further maximize the isopentenol productivity, we enhanced the precursor and NADPH supplies needed for the MEP pathway by activating the pentose phosphate pathway (PPP) and Entner-Doudoroff (ED) pathway through the disruption of the *E. coli* phosphoglucose isomerase gene (*pgi*). To evaluate the efficacy of our multi-step optimization of isopentenol production, we finally implemented a 5 L-scale laboratory batch fermentation using the optimal strain identified.

## Results and discussion

### Expression of nudF and yhfR in *E. coli* W3110

**Figure 2:**
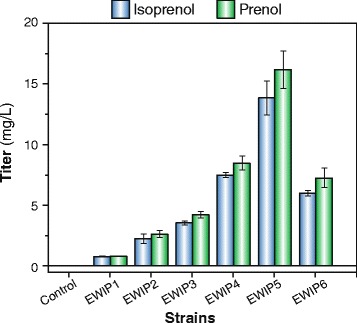
**Isopentenol production by engineered** ***E. coli*** **strains.** A 250 mL-scale shaking flask containing 50 mL of semi-defined medium, consisted of M9 minimum salts, 5 g L^−1^ yeast extract, 10 g L^−1^ glucose and 1 mM thiamine pyrophosphate (TPP), was used for the cultivation of the strains. All strains are listed in Table 1.

To enable a successful isopentenol production in *E. coli*, two genes, *nudF and yhfR* from *Bacillus subtilis*, were identified as heterologous enzyme genes for direct conversion of IPP and DMAPP into isoprenol and prenol respectively. However, due to the different codon usages of *E. coli* and *B. subtilis*, the codon adaption indexes (CAI) of the wild-type *yhfR* (GeneBank: CP002906.1) and *nudF* (GeneBank: CP003329.1) genes for the host *E. coli* W3110 are only 0.31 and 0.25 respectively [17]. To address the potential low expression issue in the *E. coli* host, the ORF sequences of these two genes were optimized to achieve a CAI of 1.0 for both (See Additional file 1). The optimized *yhfR* and *nudF* genes were then synthesized, ligated into the expression vector *pACYC-duet*, and subsequently introduced into *E. coli* W3110 (DE3) for isopentenol production. GC analysis results showed that, after 48 h of cultivation in M9 medium containing yeast extract and glucose, the strain *E. coli* W3110(DE3)/*pACYC-yhfR* produced a tiny amount of isoprenol and prenol (<1 mg L^−1^) (Figure 2, EWIP1). In contrast, the strain *E. coli* W3110 (DE3)/*pACYC-nudF* produced 2.6 mg L^−1^ prenol and 2.2 mg L^−1^ isoprenol (Figure 2, EWIP2). No isoprenol or prenol production was detected in the control strain that harbors the empty vector *pACYC-duet* (Figure 2, Control). These results supported the earlier shown feasibility of producing isopentenol in *E. coli* and also suggested that NudF is a more effective enzyme than yhfR in isopentenol production.

### Optimization of MEP pathway for increased isopentenol production

To elevate isopentenol production, the upstream MEP pathway was targeted for optimization. In a recent study, Zhou *et al.* showed that overexpression of the *E. coli* HMBPP synthase gene (*ispG*) can increase the isoprenoid production by reducing MECPP efflux, i.e., the rate of MECPP transport out of the cell [18]. Based on this finding, the *ispG* gene was cloned and ligated into *pACYC-nudF*, resulting in a plasmid denoted as *pACYC-ispG-nudF*. An overexpression experiment of the *E. coli* W3110 (DE3)/*pACYC-ispG-nudF* showed that *ispG* co-expression led to a 1.6-fold increase in isopentenol production (Figure 2, EWIP 3).

Additionally, two recent studies have shown that overexpression of the DXP synthase gene *dxs* is able to enhance the metabolic flux of the MEP pathway and hence isoprenoid production [19,20]. Encouraged by these results, we further over-expressed the *dxs* gene along with *ispG* and *nudF* in *E. coli* W3110 (DE3). Encouragingly, the new strain resulted in an additional 2.1-fold increase in isopentenol production (Figure 2, EWIP 4).

### Tuning the glycolysis pathway to maximize the isopentenol production

To further increase the isopentenol production of our engineered strains, we traced back to the precursor supplies of the MEP pathway and examined their roles in influencing the overall isopentenol production.

**Figure 3:**
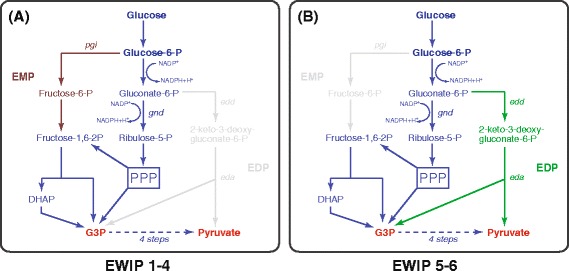
**Thee glycolytic pathways present in** ***E. coli*** **. (A)**, The native EMP and PPP pathways are active in the *E. coli* strains EWIP1-4. **(B)** The PPP and ED pathways are activated in the strain EWIP5 by disrupting the *pgi* gene. EMP, Embden-Meyerhof pathway; PPP, pentose phosphate pathway; ED, Entner-Doudoroff.

As shown in the first step of MEP pathway in Figure 1, one mole of deoxyxylulose-5-phosphate (DXP) is formed from the equimolar condensation of glyceraldehyde-3-phosphate (G3P) and pyruvate. This suggests that limitations in G3P or pyruvate or their imbalance may reduce DXP formation and hence hinder isopentenol production [21]. In wild-type *E. coli* strains, glucose is metabolized mainly through the Embden-Meyerhof-Parnas pathway (~70%) and partially through Pentose Phosphate Pathway (~30%) as depicted in Figure 3 (panel A). Both of the pathways lack the control of pyruvate and G3P productions, which may imply an imbalanced G3P-pyruvate ratio. Indeed, a couple of previous studies have supported this speculation [22,23]: They showed that re-balancing the pyruvate and G3P pools via the modification of the EMP pathway can increase the isoprenoid production significantly.

Meanwhile, there is an additional glycolytic pathway responsible for glucose metabolism in *E. coli*–the Enter-Doudoroff pathway [24,25]. The ED pathway is silent in native *E. coli* strains consuming glucose but it is capable of simultaneous production of pyruvate and G3P because the pathway generates the two precursors concurrently with a common cleavage reaction (Figure 3, panel B). Therefore, compared with the EMP pathway, the ED pathway is a potentially more appealing route for supplying the precursors of the MEP pathway.

In addition, NADPH supply is another factor that influences the efficiency of the MEP pathway. This is because two moles of NADPH are needed in order to produce one mole of isoprenoid unit (Figure 1). A recent study has indeed proven that enhancing the carbon flux to the PPP pathway, the major NADPH producing system in *E. coli*, can increase isoprenoid production [26].

Taking into account both the precursor balance and NADPH supply, we decided to shunt the glucose metabolism from EMP to PPP and ED to maximize our isopentenol production. To this end, we blocked the EMP by disrupting the *pgi* gene in *E. coli* W3110, out of the reason that *pgi* disruption can completely channel the glucose metabolic flux to PPP (~90%) and ED (~10%) from EMP [27–29]. Shaking flask cultivation experiments showed that it significantly increased the isopentenol production to 30.0 mg L^−1^ (Figure 2, EWIP5), 1.9 fold higher than the strain EWIP4.

We also replaced the native promoter of *edd-eda* operon (*eddp1*) by a strong inducible *Trc* promoter in *E. coli* W3110∆*pgi* (DE3) chromosome with the goal of altering the carbon flux between PPP and ED. However, this modification severely affected cellular growth and glucose consumption and caused a decrease in isopentenol production (Figure 2, EWIP6). One possible reason is the reduction of total NADPH production—two moles of NADPH production from the PPP pathway compared with one mole of NADPH via ED from one mole of glucose. Future studies in the impacts of EMP blockage and *eddp1* replacement are needed for further optimization of isopentenol production. In addition, a comprehensive study on the alteration of metabolic flux upon EMP blockage and *eddp1* replacement will be also valuable in order to elucidate the PPP and ED contributions to MEP pathway and isopentenol production.

### Lab-scale batch production of isopentenols

**Figure 4:**
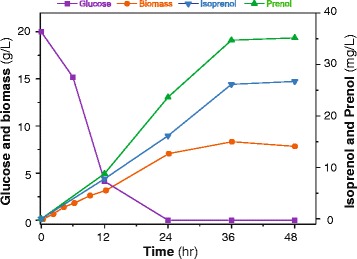
**Time course of isopentenol production, glucose consumption and biomass formation of the engineered strain** ***E. coli*** **EWIP5 during batch fermentation.**

To evaluate the outcome of our multi-step optimization of isopentenol production, a 5 L-scale laboratory batch reactor was used for fermentation. Our most optimized strain, EWIP5, was grown in 2 L M9 minimal salts medium containing 5 g L^−1^ yeast extract and 20 g L^−1^ glucose. Our fermentation results (Figure 4) showed that glucose was completely consumed at hour 24 and 23.7 mg L^−1^ of prenol and 16.2 mg L^−1^ of isoprenol were produced. However, the prenol and isoprenol levels continued to increase and reached 35.2 mg L^−1^ and 26.7 mg L^−1^ at hour 48. We speculate that the additional isopentenol production after glucose depletion was due to the consumption of yeast extract and/or other secondary metabolites (such as acetic acid) produced from glucose.

## Conclusions

In this paper, we have successfully exploited the *E. coli* native MEP pathway for the production of isopentenols including prenol and isoprenol. We chose MEP instead of MVA as the optimization target because the former is more energetically balanced and theoretically more efficient than the latter in converting sugars or glycerol to isoprenoid according to the stoichiometry and redox balance analysis [16]. To achieve our goal, multi-step optimizations were employed to increase isopentenol production: The expression of codon-optimized *nudF* in *E. coli* W3110 led to the production of both isoprenol and prenol; overexpression of the HMBPP synthase gene *ispG* and DXP synthase gene *dxs* resulted in an additional 3.3-fold increase in isopentenols production; and shunting the glycolysis from EMP to PPP and ED led to another 1.9-fold increase. Through the multi-step optimizations, the resulting strain was able to produce isopentenols at a level of 61.9 mg L^−1^.

It is also worthy to notice that the blocking of EMP to channel glucose metabolism via PPP and ED explored here provided a more balanced precursor pool for the MEP pathway. Although further studies are required to elucidate the exact contributions of PPP and ED to isopentenol production, this strategy may serve as a novel approach for further optimization of MEP-dependent isoprenoid biosynthesis. In the future, additional efforts, such as those on precursor balance and NADPH supply, are also needed for further improvement of the titer of isopentenols.

## Methods

### Strains and plasmids

All strains and plasmids used in this study are listed in Table 1. *E. coli* W3110 was purchased from ATCC (No. 27325), whereas *E. coli* W3110 ∆*pgi* was constructed by deleting *pgi* gene in it. The *pgi* gene disruption cassette was amplified with primers Pgi-KF and Pgi-KR (Table 2) using pKD3 as template. The *eddp1* replacement with *pTrc* promoter was carried out using the cassette amplified with primers Eddp1-KF and Eddp1-KR and pKD4 as template. The plasmid pKD46 was used as the Red recombinase expression vector while pCP20 was used to eliminate the resistance gene. Gene disruption and resistant gene elimination experiments were performed according to the protocols obtained from OPENWETWARE [30]. To express the relevant genes under the control of the T7 promoter, the 69734 λDE3 Lysogenization Kit (Novagen, EMD Millipore, U.S.) was used to integrate an λDE3 prophage into the *E. coli* host chromosome.

**Table 1:** **Strains and plasmids used in this work**

**Plasmid/strain**	**Function/characteristic**	**Resource/reference**
*pACYC-yhfR*	*yhfR* expression vector	This work
*pACYC-nudF*	*nudF* expression vector	This work
*pACYC-ispG-nudF*	*ispG* and *nudF* co-expression vector	This work
*pACYC-dxs-ispG-nudF*	*dxs*, *ispG* and *nudF* co-expression vector	ATCC No. 27325
W3110	*E. coli* W3110 F^−^, λ^*−*^ IN (rrnD-rrnE)1	ATCC No. 27325
∆*pgi*	*E. coli* W3110 ∆*pgi*	This work
∆*pgi* ∆*eddp1::pTrc*	*E. coli* W3110 ∆*pgi* ∆*eddp1::pTrc*	This work
EWIP1	*E. coli* W3110 (DE3)/*pACYC- yhfR*	This work
EWIP2	*E. coli* W3110 (DE3)/*pACYC-nudF*	This work
EWIP3	*E. coli* W3110 (DE3)/*pACYC-ispG-nudF*	This work
EWIP4	*E. coli* W3110 (DE3)/*pACYC-dxs-ispG-nudF*	This work
EWIP5	*E. coli* W3110 (DE3) ∆*pgi*/*pACYC-dxs-ispG-nudF*	This work
EWIP6	*E. coli* W3110 (DE3) ∆*pgi* ∆*eddp1::pTrc/pACYC-dxs-ispG-nudF*	This work

**Table 2:** **Primers used in this study**

**Name**	**Sequence (5′-3′)**	**Function**
Dxs-F	CGTCGGATCCATGAGTTTTGATATTGCCA	For PCR amplification of *dxs*
Dxs-R	CGGGAATTCTTATGCCAGCCAGGCCTTG	
IspG-F	CACGAGCTCAGGAGATATACCATGCATAACCAGGCTCCAAT	For PCR amplification of *ispG*
IspG-R	CGTGAGCTCTTATTTTTCAACCTGCTGAACGT	
Eddp1-KF	TCTGCGCTTATCCTTTATGGTTATTTTACCGGTAACATGACATATGAATATCCTCCTTAGT	For PCR amplification of *eddp1* replacement cassette
Eddp1-KF	CAATGATTCGATTTGTTACGCGTAACAATTGTGGATTCATTGTTTATTCCTCCTTACATTATACGAGCCGGATGATTAATTGTCAAGTGTAGGCTGGAGCTGCTTCG	
TrcIN-F	GTGGCGATGATTACCCGTGA	For verification of eddp1 replacement with *pTrc*
TrcIN-R	GGTTACCGCATGCCAACTGC	
Pgi-KF	CTTCTCAGAAGCGATTATTTCCGGTGAGTGGAAAGGTTATCATATGAATATCCTCCTTAGT	For PCR amplification of *pgi* disruption cassette
Pgi-KR	TACCGTTACGGTCAACATACTTACCGTTGGACTCCATATTGTGTAGGCTGGAGCTGCTTCG	
Pgi-F	TACTCCAAAAACCGCATCAC	For verification of *pgi* disruption
Pgi-R	CGAAGAAGTTAGACAGCAGT	

The codon adaption indices (CAI) of the *B. subtilis yhfR* and *nudF* genes were analyzed using Optimizer [17]. The codon-optimized sequences of *yhfR* and *nudF* were synthesized and ligated into *pACYCDuet-1* by *Nde*I and *BglII* to construct *pACYC-yhfR and pACYC-nudf,* respectively. The DXP synthase gene (*dxs*) was ligated into *pACYC-nudf* using *NcoI* and *Bam*HI, followed by further ligation of the ispG with *HindIII*. The final plasmid was denoted as *pACYC-dxs-ispG-nudF*. All primers used for the construction of plasmids are listed in Table 2.

### Culture conditions

A 250 mL shaking flask containing 50 mL of semi-defined medium, consisted of M9 minimum salts, 5 g L^−1^ yeast extract, 10 g L^−1^ glucose and 1 mM thiamine pyrophosphate (TPP), was utilized for the cultivation of the isopentenol producing strains, chloramphenicol (35 mg mL^−1^) was also added into the medium. The shaking flask was inoculated with 1 mL of overnight culture and incubated with 150 rpm agitation at 37°C. 0.5 mM Isopropyl ßD-1-thiogalactopyranoside (IPTG) was added when the optical density (OD_600_) of the culture reached 0.3 AU. After IPTG addition, the culture was transferred into 30°C shaking incubator (150 rpm) for 48 h of cultivation.

For fermenter scale isopentenol production, a 5 L laboratory reactor containing 2 L medium was used. The 2 L fermentation medium contained M9 minimum salts, 5 g L^−1^ yeast extract, 20 g L^−1^ glucose and 1 mM TPP. Chloramphenicol was also added into the fermentation medium at the same time. Inoculant was prepared by introducing a single colony picked from an agar plate into 5 mL of LB medium containing chloramphenicol. The culture was grown at 37°C with 150 rpm agitation. After 12 h, the culture was transferred to 100 mL fresh LB medium containing chloramphenicol and was further cultured for another 12 h. The inoculant was then transferred into the fermentation vessel to initiate the batch fermentation (t = 0 h). When OD_600_ value reached 3.0 AU, 0.5 mM IPTG was added and the temperature was decreased to 34°C. The agitation speed was controlled by PID to maintain the dissolved oxygen (D.O.) at 20% air saturation. Foaming was controlled by addition of antifoam A whereas the pH was maintained at 7.0 by addition of 2 N H_2_SO_4_ or NH_4_OH (28%).

### Biomass analysis

Cellular growth was measured in terms of OD_600_. For the calculation of biomass production, a standard curve of dry cell weight was correlated with OD_600_. Samples were collected in 2 mL pre-weighed pre-dried centrifuge tubes and were pelleted at 8,000 *g* for 10 minutes. After discarding the supernatant, the pellets were washed twice with distilled water and dried at 105°C. One OD_600_ unit was equivalent to 0.29 g L^−1^ of dry cell weight.

### Metabolite analysis

For glucose analysis, each culture sample was pelleted by centrifugation and the collected supernatant was analyzed in Waters HPLC equipped with a Bio-Rad Aminex HPX-87H Column (300 × 7.8 mm). The eluent (5 mM H_2_SO_4_) was pumped at a flow rate of 0.4 mL min^−1^. The column temperature was maintained at 55°C and the peaks were detected using a Waters 2414 refractive index detector.

For isopentenol analysis, each sample was prepared by ethyl acetate extraction and only the upper layer was subject to GC analysis. The GC equipped with a flame ionization detector (FID) and a CP-FFAP CB capillary column (50 m × 0.25 mm; 0.2 μm film thickness) was applied. The oven temperature was initially held at 50°C for 1 min, then raised with a gradient of 5°C/min until reaching 100°C, and finally programmed to 150°C at 25°C/min, then hold for 5 min. Nitrogen was used as the carrier gas. The injector and detector were held at 250°C and 270°C, respectively. This method can separate isoprenol and prenol efficiently, the isoprenol peak appeared at 13.9 min while the prenol peak appeared at 15.3 min.

## Additional file

Additional file 1:
**Codon-optimized sequence of** ***yhfR,*** **Codon-optimized sequence of** ***nudF.***

## Abbreviations

pgi: Phosphoglucose isomerase gene
ispG: HMBPP synthase gene
dxs: DXP synthase gene
MEP: 2-methyl-(D)-erythritol-4-phosphate
PPP: Pentose phosphate pathway
ED: Entner-Doudoroff
EMP: Embden-Meyerhof-Parnas
IPP: Isopentenyl pyrophosphate
DMAPP: Dimethylallyl pyrophosphate
MVA: Mevalonate-dependent pathway
G3P: Glyceraldehyde-3-phosphate
CAI: Codon adaption index
TPP: Thiamine pyrophosphate
IPTG: ßD-1-thiogalactopyranoside
FID: Flame ionization detector
