# Supplementary material for: MEP pathway-mediated isopentenol production in metabolically engineered Escherichia coli
Source: Microb Cell Fact. 2014 Sep 12;13:135. doi: 10.1186/s12934-014-0135-y (PMC4172795; doi:10.1186/s12934-014-0135-y)
Supplement: Additional file 1: — Codon-optimized sequence of yhfR, Codon-optimized sequence of nudF. [file 12934_2014_135_MOESM1_ESM.pdf]

Additional file 1

Codon-optimized sequence of *yhfR*

ATGACCGCGGTTTGCCTGGTTCGTCACGGTGAAACCGACTGGAACCTGCAGCAGAA
ATGCCAGGGTAAAACCGACATCCCGCTGAACGCGACCGGTGAACGTCAGGCGCGTG
AAACCGGTGAATACGTTAAAGACTTCTCTTGGGACATCATCGTTACCTCTCCGCTGA
AACGTGCGAAACGTACCGCGGAAATCATCAACGAATACCTGCACCTGCCGATCGTT
GAAATGGACGACTTCAAAGAACGTGACTACGGTGACGCGGAAGGTATGCCGCTGGA
AGAACGTACCAAACGTTACCCGGACAACATCTACCCGAACATGGAAACCCTGGAAG
AACTGACCGACCGTCTGATGGGTGGTCTGGCGAAAGTTAACCAGGCGTACCCGAAC
CAGAAAGTTCTGATCGTTGCGCACGGTGCGGCGATCCACGCGCTGCTGACCGAAAT
CTCTGGTGGTGACCCGGAACTGCAGTCTACCCGTCTGGTTAACGCGTGCCTGTCTAA
CATCGAATTCGCGGAAGAAAAATGGCGTATCAAAGACTACAACATCAACTCTCACC
TGTCTGGTTTCATCAAATAA

Codon-optimized sequence of *nudF*.

ATGAAATCTCTGGAAGAAAAAACCATCGCGAAAGAACAGATCTTCTCTGGTAAAGT
TATCGACCTGTACGTTGAAGACGTTGAACTGCCGAACGGTAAAGCGTCTAAACGTG
AAATCGTTAAACACCCGGGTGCGGTTGCGGTTCTGGCGGTTACCGACGAAGGTAAA
ATCATCATGGTTAAACAGTTCCGTAAACCGCTGGAACGTACCATCGTTGAAATCCCG
GCGGGTAAACTGGAAAAAGGTGAAGAACCGGAATACACCGCGCTGCGTGAACTGG
AAGAAGAAACCGGTTACACCGCGAAAAAACTGACCAAAATCACCGCGTTCTACACC
TCTCCGGGTTTCGCGGACGAAATCGTTCACGTTTTCCTGGCGGAAGAACTGTCTGTT
CTGGAAGAAAAACGTGAACTGGACGAAGACGAATTCGTTGAAGTTATGGAAGTTAC
CCTGGAAGACGCGCTGAAACTGGTTGAATCTCGTGAAGTTTACGACGCGAAAACCG
CGTACGCGATCCAGTACCTGCAGCTGAAAGAAGCGCTGCAGGCGCAGAAATGA
